# The enigmatic clock of dinoflagellates, is it unique?

**DOI:** 10.3389/fmicb.2022.1004074

**Published:** 2022-10-19

**Authors:** Dinesh Balasaheb Jadhav, Yoshita Sriramkumar, Sougata Roy

**Affiliations:** Department of Biology, Ashoka University, Sonipat, Haryana, India

**Keywords:** phytoplankton, dinoflagellate, circadian clock, non transcriptional, casein kinase

## Abstract

Dinoflagellate clocks are unique as they show no resemblance to any known model eukaryotic or prokaryotic clock architecture. Dinoflagellates are unicellular, photosynthetic, primarily marine eukaryotes are known for their unique biology and rhythmic physiology. Their physiological rhythms are driven by an internal oscillator whose molecular underpinnings are yet unknown. One of the primary reasons that slowed the progression of their molecular studies is their extremely large and repetitive genomes. Dinoflagellates are primary contributors to the global carbon cycle and oxygen levels, therefore, comprehending their internal clock architecture and its interaction with their physiology becomes a subject of utmost importance. The advent of high throughput Omics technology provided the momentum to understand the molecular architecture and functioning of the dinoflagellate clocks. We use these extensive databases to perform meta-analysis to reveal the status of clock components in dinoflagellates. In this article, we will delve deep into the various “Omics” studies that catered to various breakthroughs in the field of circadian biology in these organisms that were not possible earlier. The overall inference from these omics studies points toward an uncommon eukaryotic clock model, which can provide promising leads to understand the evolution of molecular clocks.

## Introduction

### The clock architecture

Circadian rhythms are present across kingdoms from cyanobacteria to humans that manifest as overt oscillation in behavior, physiology, biochemistry, and metabolism (Patke et al., 2019). These rhythms are generated by an inherent cell-autonomous clock that confer an adaptive advantage to organisms that evolved in the 24-h day/night cycle of the earth (Dunlap, 1999). Although the clock constituents vary, their core architecture remains intact. These clocks are run by underlying oscillators and to date, we are aware of 3 such different oscillators. First is the transcriptional-translational feedback loop (TTFL) oscillator (Lande-Diner et al., 2013) found mostly in eukaryotes, second is the post-translationally operated Kai oscillators (PTO) in prokaryotes (Golden and Canales, 2003), and finally, the recently revealed metabolic/redox oscillator that serves as an ancient and conserved oscillator across all lineages (Edgar et al., 2012). However, dinoflagellates are unicellular marine eukaryotes whose clock architecture fits neither the eukaryotic TTFL model nor the prokaryotic PTO model (Roy et al., 2014a). Although the presence of the conserved metabolic/redox oscillator is anticipated it has not been experimentally validated in these species.

### The dinoflagellates

Dinoflagellates are unicellular eukaryotes that are predominantly photosynthetic and marine dwellers, however, heterotrophic, mixotrophic, and freshwater species are also found (Carty and Parrow, 2015). They are known for their extensive harmful blooms (Anderson et al., 2008; Borbor-Cordova et al., 2019) and nightly bioluminescence in the ocean (Valiadi and Iglesias-Rodriguez, 2013). Some of them produce neurotoxins that are poisonous to marine mammals and humans, posing an economic threat to the fishery industry (Wang, 2008). Along with the diatoms, dinoflagellates are the primary producers that capture and fix the greenhouse gas carbon dioxide and release oxygen, which accounts for almost half of the global oxygen content (Field et al., 1998; Falkowski, 2012). The circadian clock in these marine dinoflagellates drives their rhythmic physiology such as photosynthesis and nitrogen metabolism, which regulates the marine carbon and nitrogen cycles, respectively. Therefore, understanding the inherent clock and its impact on dinoflagellate physiology becomes a topic of primary interest.

Among the eukaryotes, dinoflagellates are known for their unique molecular features, such as enormous genomes (Wisecaver and Hackett, 2010), which are packed in liquid crystalline chromosomes (Costas and Goyanes, 2005). This is unlike the nucleosome structure found in all other known model eukaryotes. *Lingulodinium polyedra* (previously *Gonyaulax polyedra*) emerged at the forefront as a model dinoflagellate species suitable to study circadian physiology. This is because *L. polyedra* has many easy and tractable overt physiological rhythms that were found to be under clock regulation (McMurry and Hastings, 1972). One such extremely interesting rhythm is that of bioluminescence (Fritz et al., 1990), an easily readable reporter mechanism that is in-built and runs under a circadian program. Vertical migration, aggregation (Roenneberg and Hastings, 1988), cell division (Vicker et al., 1988), photosynthesis (Sweeney, 1986), nitrogen assimilation (Dagenais-Bellefeuille and Morse, 2013) are the other well-known clock controlled processes. Apart from this, several proteins and enzymes also follow the circadian phase of expression (Johnson et al., 1984). However, the underlying physiological roles of these dynamics are not yet clear. Unlike the other model eukaryotes, dinoflagellates do not show extensive transcriptional regulation; rather translational control is rampant (Fagan et al., 1999; Hastings, 2007). Also, dinoflagellates have a very low abundance of transcription factors that belong to unconventional families (Bayer et al., 2012; Beauchemin et al., 2012; Roy and Morse, 2013). Taken together, studies in *L. polyedra* showed prospects of a concealed and unconventional clock that led to – “Omics” studies including the large scale meta transcriptomics, transcriptomics (Beauchemin et al., 2012; Lauritano et al., 2017), proteomics (Roy et al., 2014b; Beauchemin and Morse, 2018; Tse et al., 2018) and phosphoproteomics (Liu et al., 2012; Roy and Morse, 2014). Although, *L. polyedra* is by far the most researched species in the context of chronobiology, there are some interesting studies with a few other species. One of them is *Symbiodinium*, a species of dinoflagellates that corals host to acquire essential photosynthates. This incited studying this as a model relevant to clock regulation of physiology in the host-symbiotic system (Sorek et al., 2013, 2014, 2018).

All research conducted thus far suggests the presence of an unusual clock organization and unique oscillator components in these organisms that is worth studying. In this review, our effort is to demonstrate these distinctive features of the dinoflagellate clock by discussing the – “Omics” studies that led to the understanding of the dinoflagellate clock, its organization, and the underlying mechanism.

## Dinoflagellate genomics and the circadian clock

The genomes of dinoflagellates are remarkable in many ways. The large DNA content (about 10–200 pg) in unicellular dinoflagellates is the highest among all known eukaryotes (Lin, 2011). The dinokaryon nucleus of dinoflagellates is one of its kind among eukaryotes (Gornik et al., 2019). Unlike any other eukaryotes their DNA is not packaged into nucleosomes (Riaz et al., 2018), although histone and histone like proteins exist, their role is not yet clear. Multiple gene copies are organized as tandem repeats along the chromosomes (Bachvaroff and Place, 2008; Beauchemin et al., 2012),which are poses a considerable challenge to the genome sequencing ventures in these species (Treangen and Salzberg, 2012). However, the ground-breaking progress in third generation sequencing and *de novo* assembling techniques are proving beneficial for revealing the dinoflagellate genome architecture (Marinov et al., 2021; Nand et al., 2021). Utilizing these technological advancements, 15 dinoflagellate genomes have been sequenced (González-Pech et al., 2021) and new assemblies were generated providing deeper insights into the genome of already sequenced species (Lin et al., 2021). By far, the genus *Symbiodinium* has been at the forefront of such analysis because of its relatively smaller genomes (Lin et al., 2015) and its symbiotic role in sustaining its coral host. Recently, a free-living dinoflagellate genome of size ~7 Gbp was sequenced from *Polarella glacialis* (Stephens et al., 2020). One of the common features of these genomes is the presence of large sets of unannotated sequences suggesting either the emergence of novel gene families or excessive divergence resulting in no significant similarity with the putative original sequences (Aranda et al., 2016). Current literature on dinoflagellate genomes did not reveal any central clock components related to either eukaryotic TTFL oscillator or prokaryotic *Kai* oscillator genes (Shoguchi et al., 2013; Lin et al., 2015; Noordally and Millar, 2015). However, these studies in dinoflagellates are a major step in understanding its genome organization leading to the better realization of the underlying regulatory mechanism (Lin et al., 2015). With the availability of the genome sequences, it will be convenient to generate a robust database for downstream transcriptomics, proteomics and phosphoproteomics studies. Further, it will be beneficial in identifying the regulatory non-coding regions that play significant roles in modulating the underlying clock. This might pan out to be an approach to capture the factors that are regulated by the clock that will eventually lead to isolating the yet unknown clock components in dinoflagellates.

Some of the facts realized from these genome studies are quite interesting, such as the presence of unconventional promoter and miRNAs-based gene regulatory machinery (Lin et al., 2015), unique chromosome structure, and telomeric enrichment of genes (Lin et al., 2021). Genome annotation of *S. kawagutii* shed light on the array of redox regulatory genes (Okamoto and Hastings, 2003).This will be a stepping stone to investigate the metabolic/redox oscillator model (Edgar et al., 2012), which has not been substantiated in this class of organisms.

**Figure 1:**
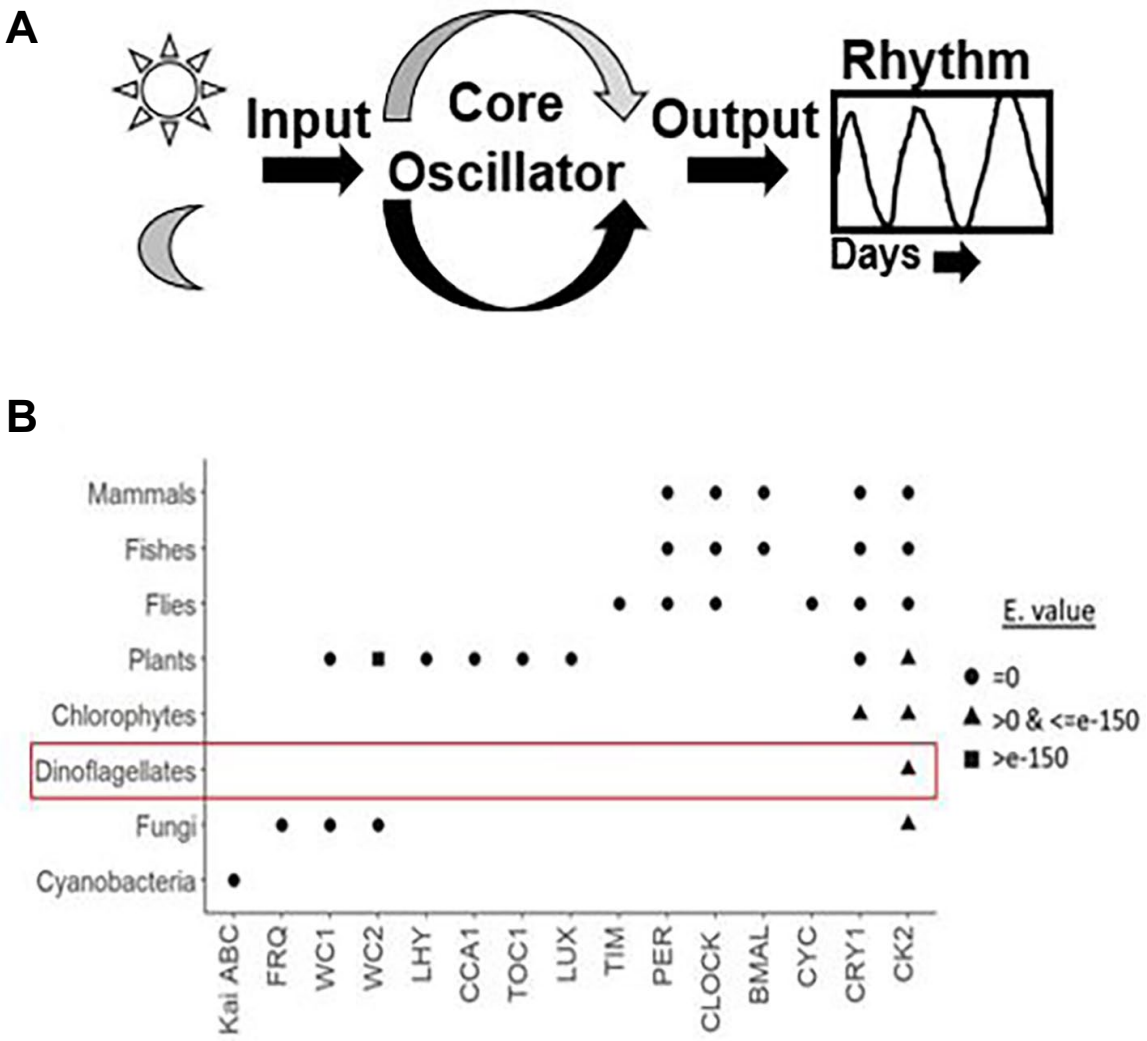
**(A)** Generalized depiction of the internal clock organization. Apart from the core oscillator, that consist of the cogs and gear that oscillate independently without the requirement of any external cues, there are cellular factors that convey the external signal to the cell-based oscillator called Inputs and others that take the information from the oscillator and then generate the overt rhythms, called outputs. Inputs synchronize the endogenous clock to the environmental time, which makes the clock flexible to adjustments to different light regimes at different time zones. **(B)** Dot plot showing an overview of all known clock components across various taxa based using the R algorithm. X and Y-axes denotes core clock proteins and model organisms, respectively. The row corresponding to each organism constitute the clock proteins present in it. The shapes represent the respective e-values. We used three approaches, firstly, for clock model organisms we directly documented the evidence from the KEGG pathway database. Secondly, for organisms that are well annotated we got their clock genes from the literature. Thirdly, for dinoflagellates annotation is not yet comprehensive. Therefore, the clock proteins from respective species were downloaded from either the KEGG pathway “circadian rhythm” subsection or the NCBI database. Each clock protein was blasted (tblastn) against the dinoflagellate comprehensive datasets in NCBI with these parameters (Query coverage - ≥50%, Percent identity - ≥50% and E value <E-10). We accept only if all the 3 criteria are satisfied. The clock proteins found in dinoflagellates are encircled, note the presence of Casein kinase in all known eukaryotes.

Using the comprehensive genome information, clock components in different species have been identified. A lineage wise description of clock components and their distribution across species is portrayed in Figure 1B. The Casein kinase (CK) family stands out to be the sole conserved link between dinoflagellate and mammalian clocks. Although CKs have a major role in clocks, they are also recognized for their pleiotropic functions as an essential kinase (Issinger, 1993; Franchin et al., 2018).

## Transcriptomic studies revealed interesting features of the dinoflagellate circadian clock

The emergence of Next Generation Sequencing (NGS) opened the otherwise refractory dinoflagellates to functional genomics studies (Goodwin et al., 2016). The advent of NGS and its widespread utilization in dinoflagellates is described elsewhere (Guo et al., 2016). In this section, we will focus on the transcriptomic studies that contributed significantly to understanding the unusual circadian systems of dinoflagellates.

RNA-seq is now a gold-standard technique to not only profile the entire RNA population within the cells (Wang et al., 2009) but also reveal the transcriptome-wide dynamics of RNA across the circadian cycle (Zhang et al., 2014). The technological advancement of RNA-seq allows investigation of all the major RNA subtypes, however, studying mRNA dynamics has been prioritized due to obvious reasons. RNA-seq has been the most sought-after technology to quantify the changes in mRNA abundance across the day/night cycle (Li et al., 2015), a measure to demonstrate the extent of clock regulation on the global transcription in eukaryotes with a TTFL oscillator (Roenneberg and Merrow, 2005). The general consensus with TTFL oscillators is that they impart the daily regulation of physiology, biochemistry, and metabolism primarily through rhythmic RNAs (Pizarro et al., 2013) that are then expected to generate an equivalent downstream rhythms in the respective proteins. With the incorporation of a high-throughput analysis component, it is now possible to analyse the circadian post-transcriptional events such as splicing from the transcriptome datasets (Romanowski and Yanovsky, 2015).

Several studies led to the fact that circadian regulatory mechanisms can modulate post-transcriptional mechanisms to impart daily changes in physiology and biochemistry (Mauvoisin, 2019). Some of these interesting observations came from dinoflagellates. *L. polyedra* is the first model system where rhythms in protein synthesis and degradation were shown to propagate without any significant changes in their corresponding mRNA levels (Morse et al., 1989). *L. polyedra* demonstrate daily rhythms in bioluminescence (Valiadi and Iglesias-Rodriguez, 2013). The biochemistry underlying the circadian regulation of bioluminescence exhibited a clear role of temporal regulation in protein synthesis without any involvement of transcriptional regulation (Milos et al., 1990). Luciferase (LCF) and luciferin binding protein (LBP) are the only two proteins involved in the regulation of bioluminescence. Both LCF and LBP showed nightly expression of proteins, however, their mRNA levels are constant across the day-night cycle. A transcriptome-wide RNA abundance analysis across the daily cycle would exhibit the extent of transcriptional control in these unconventional eukaryotes. With this aim, we carried out a transcriptome-wide RNA-seq study in the dinoflagellate *L. polyedra* and found no significant changes at the transcription level across the circadian cycle. Additionally, drug-mediated inhibition of transcription does not affect the bioluminescence and pH rhythms, which are well-known readouts of the underlying clock (Roy et al., 2014a). This study demonstrates that RNA rhythms are not required to generate circadian rhythms in *L. polyedra*, a hallmark of the non-TTFL mode of circadian regulation. The fact that these organisms lack nucleosomes and contain low and uncommon transcription factors further supports the notion of non-transcriptional regulation in these organisms (Beauchemin et al., 2012; Roy et al., 2018). However, we did find conserved RNA transcripts of all core histones and their modifying enzymes without any traces of their proteins thereby reflecting the unconventional function of histone proteins (Roy and Morse, 2012).

Similar indications in other dinoflagellates suggested a lack of transcriptional regulation in these species. Microarray analysis revealed only 3% of *Pyrocystis lunula* (Okamoto and Hastings, 2003) and 0.7% of *Karenia brevis* (Lidie and Van Dolah, 2007) of the total transcriptome varied by 2 fold in light/dark and constant light regimes. Furthermore, comprehensive transcriptome -wide analysis of mRNA half-life in the dinoflagellate *Karenia brevis* showed a median of 33 h (Morey and Van Dolah, 2013). Similarly, long half-life were also observed for the clock regulated LUC and LBP transcripts in *L. polyedra* (Rossini et al., 2003). Circadian regulation in RNA rhythms would mean shorter half-lives of RNA that would lead to generate daily oscillation at transcript levels. Therefore, long half-lives of RNAs in dinoflagellates would rather suggest a non-transcriptional mode of regulation (Rossini et al., 2003).

On the other hand, circadian regulation of transcription is widespread in eukaryotes and for more than a decade, has been considered the primary mode of regulation that resulted in daily overt rhythms in physiology. Circadian regulation of RNA abundance can range from 10% of the total genes in *Arabidopsis* (Harmer et al., 2000; Schaffer et al., 2001), a well-known TTFL model, to as much as 65% in *Synechococcus elongatus*, a post-translational clock model (Markson et al., 2013). Although cyanobacterial circadian systems run on a non-TTFL based oscillator, daily rhythms in RNA abundance seems common (Johnson et al., 2011, 2017). It is now known that the post-translational oscillator drives the circadian changes in genome compaction that leads to these transcriptional rhythms (Markson et al., 2013). Therefore, the absence of daily rhythms in mRNA levels seems to be a unique feature of the *L. polyedra* circadian system (Roy et al., 2014a).

## Meta-analysis of consensus clock components in dinoflagellates

**Figure 2:**
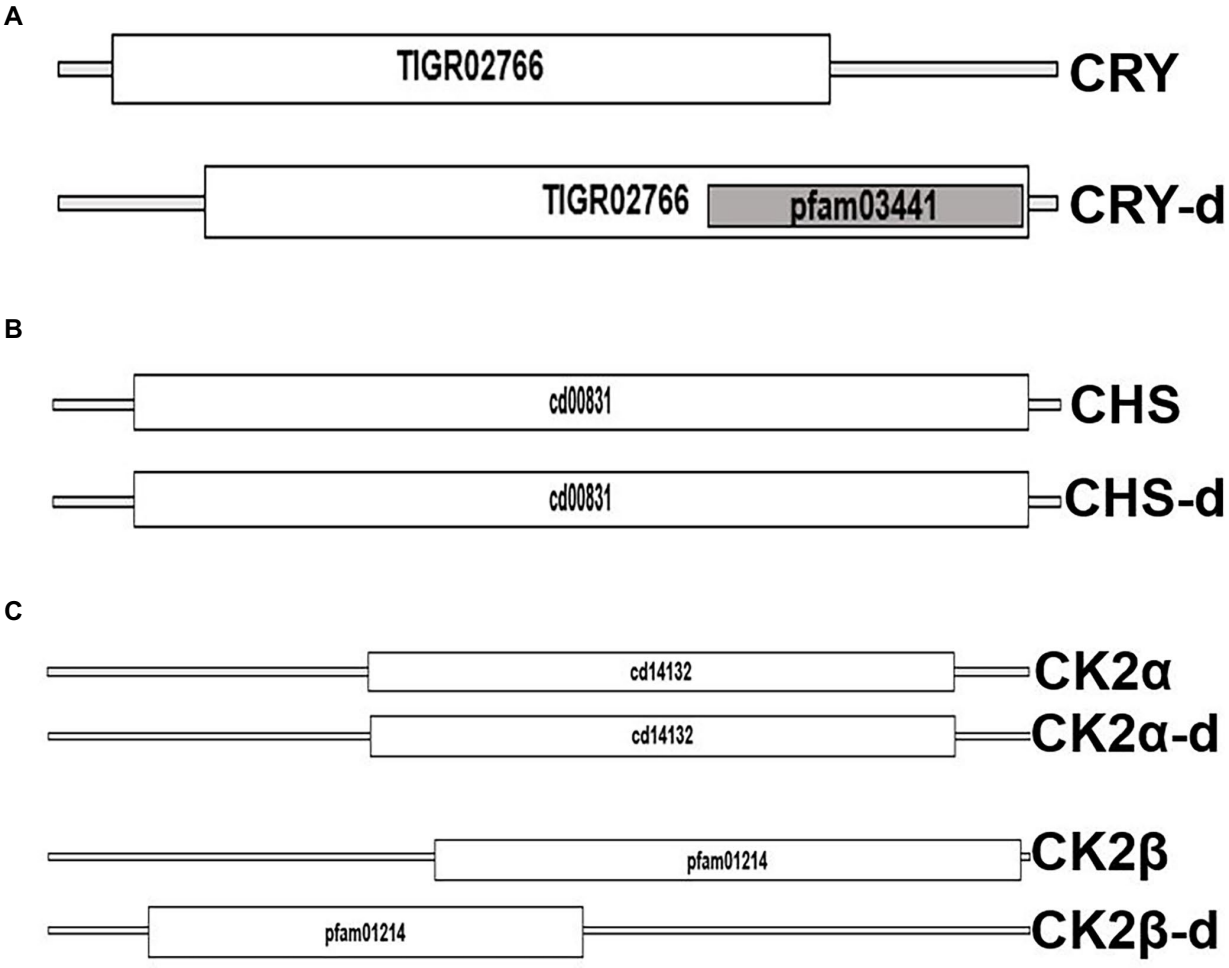
Domain level comparison of known plant clock proteins in dinoflagellates. The reference proteins were obtained from the Plant circadian rhythm sub-section of the KEGG pathway database. “-d” denotes respective proteins from dinoflagellates **(A)** Cryptochrome 2, the clock input protein, which is the blue light receptor and denoted as CRY, **(B)** Chalcone synthase, CHS, the first enzyme in flavonoid biosynthesis, and **(C)** the CK2 alpha and beta subunits known to phosphorylate the core clock proteins that fine tunes the 24-h timing of the clock.

We took advantage of the extensive transcriptome shotgun assembly (TSA) datasets of the dinoflagellate taxa currently available in the public domain and compared their homology to the plant circadian clock proteins. There are 2 reasons for selecting only plant clock proteins. First, among the photosynthetic eukaryotes, plant clock is highly annotated. Secondly, during our preliminary search of dinoflagellate TSAs we did not find any representation from other model clock species. We found 9 plant clock proteins out of the total of 26, showing some similarity to their dinoflagellate relatives (Table 1). This homology is noticed in all parts of the clock, such as input, central oscillator, and other accessory proteins. However, careful consideration is essential while inferring functions from homology driven identities. For example, a single protein (GISR01012712.1) from the dinoflagellate *Karenia mikimotoi* matches all PRR proteins from plant. All plant PRR proteins bear a high level of homology within themselves and *K. mikimotoi* having a single representation of PRR matches to all of them with different degrees of homology. On further investigation with blastn we found this sequence identical to a PRR from Oryza sativa with an E-value of e-164 (with 100% query coverage and percent identity). Therefore, to avoid such misinterpretations we further included another round of stringent conditions and selected only those candidates that have representation among at least two dinoflagellates genera (from the 93 datasets available in the public domain) with equivalent E-value, % identity and query coverage. Using these criteria, from Table 1, finally we could only select three prospective plant clock components that are represented in dinoflagellates, Cryptochrome (CRY), Chalcone synthase (CHS) and Casein kinase 2 (CK2). To get further insights, we finally conducted a domain level comparison of the three selected dinoflagellate proteins that shows considerable identity to their plant clock counterparts (Figure 2).

**Table 1:** Plant clock proteins in dinoflagellates.

	Best E-value	Species name	Accession number(s)	Comments
PHYA	Nil	Nil	Nil	
PHYB	Nil	Nil	Nil	
CRY	7.00E-105	*Symbiodinium* sp. A4 strain	GFPM01002949.1	
PIF3	Nil	Nil	Nil	
COP1	8.00E-71	*Prorocentrum micans*	GHTZ01305094.1	The query conditions are satisfied only in one species among 93 databases queried
ELF3	Nil	Nil	Nil	
SPA1	Nil	Nil	Nil	
CDF1	Nil	Nil	Nil	
FKF1	0.00E+00	*Lingulodinium polyedra*	GABP01114163.1	Same sequence is identified for two different clock proteins from the plant. The query conditions are satisfied only in one species among 93 databases queries.
HY5	Nil	Nil	Nil	
PAP1	Nil	Nil	Nil	
CO	Nil	Nil	Nil	
CHS	7.00E-154	*Lingulodinium polyedra*	GABP01095683.1	This enzyme is involved in first step of flavonoid biosynthesis
FT	Nil	Nil	Nil	
CK2α	0.00E+00	*Prorocentrum donghaiense*	GHMW01323001.1	
CK2β	7.00E-96	*Prorocentrum donghaiense*	GHMW01201102.1	
PRR3	Nil	Nil	Nil	
PRR5	Nil	Nil	Nil	
PRR7	Nil	Nil	Nil	
PRR9	Nil	Nil	Nil	
GI	2.00E-143	*Karenia mikimotoi*	GISR01014704.1	100% identical to Oryza sativa XM_015794097.1
ZTL	2.00E-155	*Lingulodinium polyedra*	GABP01114163.1	Same sequence is identified for two different clock proteins from plant. The query conditions are satisfied only in one species among 93 databases queries.
TOC1	Nil	Nil	Nil	
CHE	Nil	Nil	Nil	
LHY	1.00E-27	*Symbiodinium* sp. CCMP2430	HBTH01070647.1	Query coverage of only10% and same sequence is identified for two different clock proteins
CCA1	6.00E-27	*Symbiodinium* sp. CCMP2430	HBTH01070647.1	Query coverage of only 10% and same sequence is identified for two different clock proteins

The Plant circadian clock proteins from KEGG pathway database have been used as a query to find equivalent clock genes in dinoflagellates using tblastn (NCBI). Only those dinoflagellates have been considered whose either query coverage is ≥50%, percent identity is ≥35% and have an E-value of ≤1e–05. The dinoflagellate sequences that are not considered as putative match to their plant counterparts either have multiple hits and/or are present only in one dinoflagellate species (among the 93 datasets) as mentioned under the comments column.

From the public domains, we were able to retrieve full length sequences of CRY, CHS, and CK2 from dinoflagellates. Their domain looks identical to the plant counterparts; however, their respective protein length and domain positioning differ (Figure 2). CHS is a rate-limiting enzyme for the flavonoid biosynthesis pathway (Grotewold, 2006; Saito et al., 2013). Transcriptional dynamics of central clock components (such as CCA1 and LHY), as well as diurnal physiological rhythms in *Arabidopsis,* is altered in CHS null mutants (Hildreth et al., 2022). This is an indication of the involvement of downstream flavonoids in clock regulation, which is consistent with the recent realization of a conserved metabolic/redox oscillator across species. Among the three, CK2 is the conserved central clock component present across all the known eukaryotic circadian model systems (Figure 1), therefore, its presence in dinoflagellates suggests its important role in regulating this unique clock. However, the experimental validation of its importance as a central clock element in the dinoflagellate clock is still awaited. We have provided some indirect evidence of CK2’s importance in this *L. polyedra* clock by studying the effect of cold shock in these cells. Cold stress induces cyst formation and stalls the clock in *L. polyedra*, however, transcriptome-wide comparison reveals no global changes in the nuclear-encoded transcript levels of cysts compared to motile cells (Roy et al., 2014b). However, we found a considerable variation when comparing the phosphoproteome profile in the cyst to normal cells. It seems the phosphorylation/dephosphorylation dynamics play a significant part in regulating the dinoflagellate clock. Further, *in silico* analysis showed downregulation of phosphorylation in CK2 targets (Roy et al., 2014b), an interesting feature that needs further investigation.

## Advancement in proteomics and circadian clock research in dinoflagellates

Proteins mediate almost all the physiological processes in the cells. Temporal changes in physiology are governed by the underlying protein levels and their activity, which is regulated by the clock (Dunlap, 1999). Although regulation of RNA synthesis is considered as the predominant clock-controlled mechanism, recent studies showed a considerable contribution from daily regulation of post-transcriptional mechanisms (Zhao et al., 2004; Hastings, 2007; Kojima et al., 2011; Romanowski and Yanovsky, 2015; Green, 2018). Almost half of the mammalian circadian liver proteome showed daily changes in the proteome level without any changes in the daily mRNA levels (Wang et al., 2018). The importance of circadian regulation at the level of protein synthesis was first discovered in the *L. polyedra* bioluminescence system (Morse et al., 1989). *L. polyedra* emerged as a unique model where clock regulation of protein synthesis was found to be rampant. Some evident examples are circadian rhythms in protein abundance and activity of an enzyme of the tricarboxylic acid (TCA) cycle, NADP-dependent isocitrate dehydrogenase (NADP-ICDH) (Akimoto et al., 2005), in a glycolytic pathway enzyme glyceraldehyde 3-phosphate dehydrogenase (GAPDH) (Fagan et al., 1999), in an antioxidant superoxide dismutase (Okamoto et al., 2001) known to mitigate cellular redox stress. Although the synthesis of peridinin-chlorophyll *a*-binding protein (PCP), the protein binding to the unusual peridinin pigment, is rhythmic (Ten Lohuis and Miller, 1998), the total protein abundance across the 24 cycle remains constant. Temporal regulation of protein abundance is an output of the interplay between protein synthesis and degradation (Wang et al., 2005). In the case of PCP, although the synthesis is under temporal regulation, its degradation might not be under any temporal schedule. Therefore, it is quite difficult to ascertain the physiological importance of temporal regulation of PCP synthesis. These studies suggested the possibility of a widespread role of post-transcriptional regulation in dinoflagellates that required further systematic studies. The clock regulation of protein synthesis in *L. polyedra* encouraged extensive studies in this species. 35-S methionine labeling of newly synthesized proteins in a pulsed chase experiment followed by two-dimensional gel electrophoresis revealed that synthesis of 13 proteins is regulated by the clock while their respective mRNA levels remained constant throughout the daily cycle (Milos et al., 1990). The technological advancement led to the high-throughput and highly sensitive liquid chromatography coupled to tandem mass spectrometry (LC–MS/MS) approaches that opened avenues to quantify temporal changes in protein abundance (Angel et al., 2012). A major constraint in such proteome-wide studies is the requirement of extensive databases to map the sequenced proteins. Due to the lack of comprehensive sequence databases, high-throughput proteomics studies were not possible in dinoflagellates. However, the current ground-breaking technological progress allowed the sequencing of genomes and transcriptomes of few dinoflagellate species resulting in several high quality databases. This created the opportunity of in-depth mapping of sequenced proteins (Tse et al., 2018). Further, LC–MS/MS has also been used to identify proteins from 2D-gel electrophoresis. In one such experiment, a total of 28 proteins were identified and categorized into three phases, early evening, night, and midnight (Akimoto et al., 2004). This allowed grouping of these proteins as per their temporal abundance.

**Figure 3:**
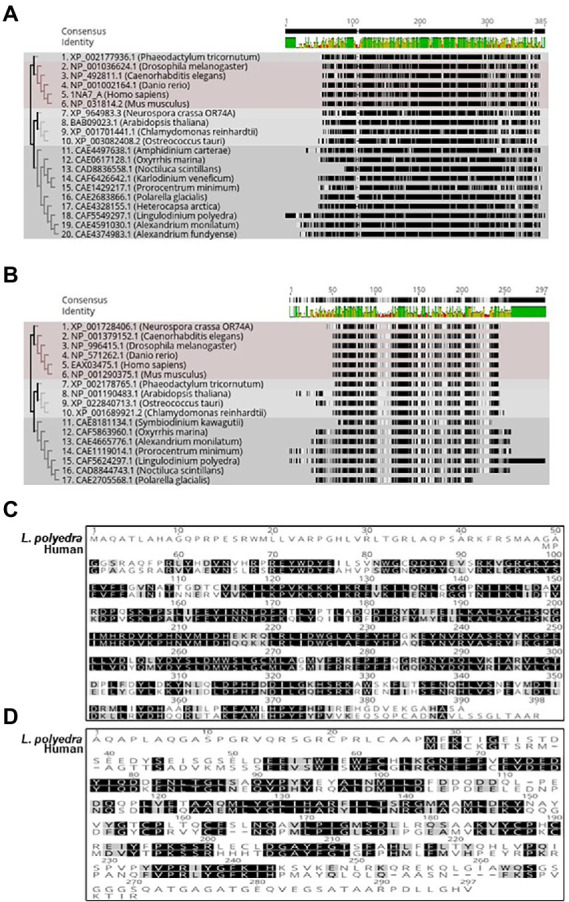
Phylogenetic tree of Dinoflagellate CK_2 constructed using the ML method embedded in RaXML software plugin in the Geneious Prime 2022.2.2 version software. Accession numbers of the sequences used to construct the tree are mentioned at the beginning of the organism’s name. **(A)** CK-2 alpha domain. **(B)** CK-2 beta domain. **(C)** Multiple sequence alignment of *Lingulodinium polyedra* CK2 alpha with human CK2 alpha using the clustalW extension in geneious prime software. **(D)** Multiple sequence alignment of *Lingulodinium polyedra* CK2 beta with human CK2 beta using the clustalW extension in geneious prime software.

The protein kinases play a significant part in modulating the internal clocks. CK2 is a well-known kinase that phosphorylates core clock elements thereby regulating their function. The finding of CK2 in dinoflagellates opened new avenues to investigate the prospective role of CK2 as a clock component in this unique clock model. However, CK2 is also a well-known and essential kinase for organisms that has vital roles outside the clock (Issinger, 1993). CK2 protein consists of a dimer of alpha and beta – subunits (Chantalat et al., 1999) where alpha confers the catalytic and beta imparts the regulatory functions (Tsuchiya et al., 2009). Phylogenetically, CK2 are widely distributed across the dinoflagellate species and represent distinct clades when compared with the other lineages (Figures 3A,B). Close comparison of the dinoflagellate CK2 α and β domains to that of the humans show key conserved regions in both proteins. Overall CK2 α seems to share a significantly higher level of sequence conservation (67% identity) to humans suggesting functional conservation in their catalytic activity (Figure 3C). In comparison, the regulatory β domain of *L. polyedra* is 45% identical to its human counterpart, suggesting the possibility of species-specific mode of regulation in dinoflagellates. However, we found few key regions that are identical, one such peptide sequence is ‘LYGLIHARYI’ that remains conserved between β domain of human and dinoflagellate counterparts (Figure 3D), whose implication remains to be addressed. The increasing number of high quality genomic and transcriptomic datasets coupled to incessant development in mass spectrometry technology will further drive the proteomics research in dinoflagellates.

## Phosphoproteomics in dinoflagellates clock research

Rhythmic changes in the protein abundance provide insights into the circadian clock regulation at the translational level. Having said that, daily dynamics at the protein levels are not enough to interpret the protein activity. Reports in a phylogenetically distant algae *Chlamydomonas reinhardtii* showed that although the amount of protein remains constant throughout the daily cycle, its activity varies during the day and night (Zhao et al., 2004). One possible interpretation is the post-translational modifications of proteins that can alter their activity. Various post-translational modifications like phosphorylation (Choudhary et al., 2015), glycosylation (Shental-Bechor and Levy, 2008), methylation (Lee et al., 2005), acetylation (Christensen et al., 2019), ubiquitylation (Wang and Wang, 2021), etc. play key roles in multiple biological processes. Phosphorylation plays a vital role in regulating the circadian clock. Enzyme CK2 is known to phosphorylate the clock protein period 2 (Lee et al., 2004). Inhibition of CK2 by DMAT increases the period length and reduces the amplitude of the daily gene expression rhythms (Tsuchiya et al., 2009). Like other organisms, the dinoflagellate *L. polyedra* clock stops when treated with a phosphorylation inhibitor 6-dimethylaminopurine (6-DMAP) (Comolli et al., 1994). By monitoring the clock driven bioluminescence rhythm, it was shown that 6-DMAP induces dose-dependent delays in the clock (Comolli et al., 1994). A follow-up study showed type 1 phosphoprotein-phosphatase as a possible regulator of circadian rhythms in *L. polyedra* (Comolli et al., 2003). This suggested that phosphatase could be a key constituent of the circadian oscillator not only in dinoflagellates but also in other organisms where clock exists (Robles et al., 2014). The dose dependent inhibition of kinases affects the clock functioning (Comolli et al., 1994), suggesting that dynamic changes in phosphoproteins are important to optimize the clock. Across different systems phosphorylation-dephosphorylation rhythms has been recognized to be one of the key mechanisms fine-tuning the inherent circadian clock (Robles et al., 2017). Even after knowing the importance of phosphorylation, the studies in dinoflagellates was limited to a few proteins due to technical limitations. The imperative need to study the global phosphoproteome dynamics led to the development of improved mass spectrometry and analysis tools.

These improved approaches were used to study the daily changes of the phosphoproteome in the dinoflagellate *L. polyedra.* In this study by Liu et al., 2012, *L. polyedra* was grown in 12 h light and 12-h dark, cells were collected every 4 h across the 24-h cycle. The cells collected from each of these timepoints were homogenized and the crude extracts were resolved on SDS-PAGE after every 4 h and stained with ProQ Diamond, a phosphoprotein specific stain. Phosphoproteome profiles differs between LD 6 and LD 18, the time when rhythmic photosynthesis and bioluminescence peaks, respectively. Protein extracts from LD6 and LD18 cells were resolved using 2-dimensional gel electrophoresis. During the day (LD6) 47 protein spots were differentially stained from the night (LD18). Consequently, 34 proteins from the night phase cells were differentially stained when compared to the day phase gels. Spots of these differentially stained proteins were picked and were identified using the TOF mass fingerprinting. Although this study provides some information, the quality and quantity of the data was below expectation (Liu et al., 2012). However, a second modified phosphoproteome study from two out-of-phase time points yielded 10-fold more phosphoproteins. The analysis of the data also revealed that many RNA binding proteins were enriched as phosphoproteins and vast majority of them have a predicted CK2 binding site (Roy and Morse, 2014). We got a similar indication from studying the cold induced temporary cysts of *L. polyedra* cells (Roy et al., 2014b). The clock stalls in the cold induced cysts, which was verified by monitoring the bioluminescence rhythms and the rhythmic LBP levels by immunoblotting studies. Transcriptome and proteome-wide comparison of cysts and motile cells showed no significant changes, whereas there was significant downregulation of phosphorylation in proteins with predicted signature of CK2 binding sites (Roy et al., 2014b). Although, concluding that CK2 has a crucial role as a core clock component in *L. polyedra* is a bit of oversimplification at this stage, these studies surely present an ideal platform to further investigate this scenario in dinoflagellates.

## Conclusion

Although there seems to be a huge diversity among clock components across eukaryotic lineages, the core TTFL or the transcriptional oscillators remains conserved. On the other hand, prokaryotes that are driven by a post translational oscillator still show substantial transcriptional regulation. Therefore, clock regulation of transcription has always captured the centre stage for understanding circadian dynamics. The dinoflagellate is emerging as a model that demonstrates the properties of a unique clock, not only in the context of the clock components but also at the level of core TTFL mechanism. This clock can function without the requirement of transcriptional dynamics, a unique feature that can lead to the discovery of a novel clock oscillator mechanism. Dinoflagellates have CK2, a kinase and a known clock protein. Although, molecular phylogeny assigns dinoflagellate CK2s to a separate clade, its catalytic domain shows 66% identity to the CK2 from human. The rampant regulation of protein synthesis, presence of low and uncommon transcription factors and presence of CK2 indicate toward a novel clock where RNA-binding proteins and posttranslational mechanisms could have a crucial role.

## Author contributions

SR: conceived the overall idea and wrote the manuscript. DJ: produced the Figure 3 and did initial drafting of the manuscript. YS: produced the Figure 1B and provided comments on the manuscript. All authors contributed to the article and approved the submitted version.

## Funding

Funding from SERB (with File Number: SRG/2019/000364) and Ashoka University’s annual research funding to SR is acknowledged. DY and YS acknowledge their PhD fellowship funding from Ashoka University.

## Acknowledgments

We thank Shivani Krishna for her comments on the manuscript.

## Conflict of interest

The authors declare that the research was conducted in the absence of any commercial or financial relationships that could be construed as a potential conflict of interest.

## Publisher’s note

All claims expressed in this article are solely those of the authors and do not necessarily represent those of their affiliated organizations, or those of the publisher, the editors and the reviewers. Any product that may be evaluated in this article, or claim that may be made by its manufacturer, is not guaranteed or endorsed by the publisher.
